# Fluid Extracts vs. Tinctures

**Published:** 1887-02

**Authors:** H. S. Lott

**Affiliations:** Augusta, Ga.


### FLUID EXTRACTS vs. TINCTURES.

BY H. S. LOTT, M. D., AUGUSTA, GA.

Some facts in regard to these two classes of preparations have occurred to me, which, in the present progressive age of our science, I feel it my duty, as it is my pleasure, to place before the medical profession.

The practitioner of medicine who realizes and appreciates the duties incumbent upon his office and the relation he bears to humanity, affording him, as it does, the means by which to relieve its sufferings, is not content to say, "let well enough alone," and thus move on throughout his career in the old grooves of his predecessors, if the "new way" is really an improvement on the old, or suggests to his mind the probability of its being worthy of consideration and trial.

What is a fluid extract, and what does the term convey to us in administering remedies which may be rendered most available in this form? As defined in our United States Pharmacopœia, "fluid extracts are permanent concentrated solutions of vegetable drugs, made of such strength that one fluid ounce contains the medicinal principles and represents the virtues of one troy ounce of the drug." To extend the definition and make it of more practical value to the prescribing physician, one *minim* of the fluid extract is equal in strength and virtue to one *grain* of the drug, possessing the great advantage, which is universally conceded, of being a solution of the active and freed in a great measure from the inert principles of the crude drug. Therefore, in prescribing a fluid extract, either separately or in combination, we give in such menstruum as to render our agent more palatable, and at the same time more efficacious, a solution of the active principles of the drug we desire to use, from which preparation of the drug we might positively expect to obtain identically the same physiological effect as that which experiments by our most careful and competent observers, and long experience by ourselves and predecessors have taught us to expect from the administration of the same agent in its original state.

Thus you see we gain at a bound, as it were, the object for which both medical men and pharmacists have so long been striving. No doubt some skeptical readers will say, have we not the same available preparations in the form of tinctures, and do we not, as we have in the past, obtain from these the desired results? To the first of these questions I answer, conscientiously, no. To the second, I will give more consideration in due time, and hope to meet the objections which will naturally arise, as they have been met and overcome in my own mind.

Are tinctures as available to the prescribing physician and as conveniently used as fluid extracts? Tinctures are "alcoholic solutions of medicinal substances;" as such, and by virtue of the necessarily various processes by which they are obtained, they cannot be made of uniform strength—uniform in the sense which we, as prescribing physicians, would have them. For instance: one tincture will represent 20 per cent. by weight of the strength of the drug and another will represent only five per cent. And why must this be the case? Because the nature of the plants has willed it so. One plant holds its active medicinal principles in a readily soluble state. As soon as the alcoholic or ethereal menstruum is poured upon the powdered drug, it yields up its active virtues and only a residuum of inert substance is left, while another plant contains a much smaller per cent. of the active principles, or becomes exhausted by the menstruum less readily. Thus you see how utterly impossible it is for us to have, in accordance with the justly fixed laws of the pharmacopœia, our tincture of a uniform strength, representing relatively minim for grain; whereas, the same facts which militate against the use of tinctures recommend fluid extracts as possessing advantages which are beyond question, and the nature of these facts is such that their truth will be at once recognized by all intelligent and practical observers.

In the manufacture of fluid extracts—and in this I have the high authority of Parke, Davis & Co. and E. R. Squibb—the relative connection between the drug and the menstruum is considered well, and demonstrated by experiment, before the process of exhaustion is begun. Such menstruum is selected as will most thoroughly exhaust the drug of its active medicinal principles, and having obtained these, free from inert and deleterious substances, will preserve them in a pure and healthy state for an indefinite space of time.

When a specified quantity of the drug has been exhausted, it is ascertained by an accurate analysis of the product what per cent. of the active principle it contains. If the product is below the standard of fluid ounce for troy ounce, minim for grain, it is raised to the same by evaporating off a portion of the menstruum and adding the required per cent. of the active principles of the drug which have been preserved from a similar process. On the other hand, if the nature of the drug is such as to place the product above the required standard—the same being proven by a similar analysis—its strength is reduced by dilution, thus accomplishing the uniformity of strength which is of such vast importance in the preparation of our medicinal substances, and giving to us a fixed law by which to be governed in formulating our prescription.

As to the mode of administering fluid extracts, I am well aware that many and seemingly just objections will be raised, especially in regard to those which are administered in minute doses or to children. But if I was as sure of all my readers being open to conviction as I am that I possess the means of convincing them, I should rest content in the eventual precedence of fluid extracts over tinctures. It has been my privilege to study, practically and closely, the manipulation of fluid extracts, and I state, without hesitation, that they are as convenient and easy to administer in minute doses, either separately or in combination, as tinctures. You say that there will be a precipitation of the resinous matter if you prescribe a fluid extract—aconite, for instance—alone, simply using water as a vehicle. I say there *will not*, in proof of which I have on my desk at this moment a mixture which has been standing for several days, formulated thus:

℞ Fld. ext. aconit. rad.....m. viii
Aqua pura............℥i
*M.*

and it stands as perfect a mixture now as when first prepared. On the addition of the water to the fluid extract there occurs a slight—very slight—milky appearance, which, however, is of no importance whatever, and, in fact, is no more than will occur on the addition of water to any of the tinctures.

If it is desired to give aconite, or any other agent used in minute doses in a combination, there are various menstrua which may be used. Of these I have found glycerine to be the best as well as the most palatable. In pneumonitis, for instance, or any disorder in which the laxative effect of the glycerine is not contraindicated, if you desire to administer a febrifuge, you will find the following most efficacious for an adult:

℞ Fld. Ext. aconit. rad..m. xxxii

Spts. ætheris nitrosi...ʒii

Glycerinæ Q. S. *ad*....℥ii

M. S. teaspoonful, which makes a mixture beautifully clear, permanent and very palatable—three advantages which are well worth our consideration.

In administering astringents, the advantages possessed by fluid extracts over tinctures are twofold. First, the astringent properties of the drug, being held in perfect and permanent solution in a practically inert menstruum, are in such a form as to come into more direct contact with the mucous surface upon which we desire them to act, thus accomplishing our object more promptly and proving more satisfactory both to our patients and ourselves. Second, when we are administering an agent of which it is necessary to give large doses, as from one to four fluid drachms, we give and obtain the result of our agent alone, and do not load the system with alcohol, which is objectionable both from a medical and a moral standpoint, but which, from the nature of tinctures, is unavoidable.

In equal manner as these advantages apply to astringents do they apply to each of the seventy-seven officinal preparations of the class termed fluid extracts, which we have laid down in our pharmacopœia, its object being to give us our medical agents in the most concentrated, permanent and palatable form.

As tonics and alteratives, we find fluid extracts equally available and convenient—a fact which, I believe, is even now pretty generally conceded. In their administration, if it is necessary to reduce their strength, and you prefer, as I do always, to make the directions a teaspoonful rather than so many drops—for very few patients will drop medicine accurately—use glycerine or equal parts of glycerine and water as a diluent, and you will obtain a mixture which is permanent and palatable.

And now finally, but in nowise of least importance, do we obtain the same therapeutic effects from the administration of fluid extracts which we have learned to expect from the use of tinctures? To my mind this question only suggests itself as one which will very naturally arise, and probably create some doubts in the minds of my readers, and not as one which will be difficult to meet, and answer as satisfactorily to such doubting minds, as it has been affirmatively decided, both by experience and observation, in my own. We may not only expect an equally good, but a better result from the therapeutic application of fluid extracts, to prove which statement, it is only necessary to recapitulate the advantages which have been set forth in the foregoing remarks. We have in fluid extracts concentrated and permanent solutions of the medicinal virtues of the drug, dissolved and perfectly suspended in such menstruum as is practically inert, and involves no danger of decomposition, from which we may positively expect to obtain the direct effect of our agent, in its pure, unadulterated state, upon the system. Let us, for example, take ergot of rye, which is unquestionably one of our most valuable medicinal agents. What physician of the present day, who has kept pace with his profession, would think of administering the tincture of ergot as oxytocic, or to relieve congestion of the spinal cord, or pulmonary hæmorrhage, when long experience and observation have proven so conclusively the superior value of the fluid extract that the tincture has become almost entirely obsolete?

Thus, both theoretically and practically, the subject of fluid extracts as a class of preparations which are superior to tinctures, not only in convenience of administration, but in their physiological and therapeutical effects, presents itself for your consideration. To those who do consider and possibly accept my views, I extend my best wishes for the success of their adoption, and to those who do not I extend a wish equally sincere in behalf of their continued use of the old class of preparations.

